# Collective violence and attitudes of women toward intimate partner violence: Evidence from the Niger Delta

**DOI:** 10.1186/1472-698X-9-12

**Published:** 2009-06-09

**Authors:** Diddy Antai, Justina Antai

**Affiliations:** 1 Division of Global Health & Inequalities, The Angels Trust – Nigeria, Abuja, Nigeria; 2 Division of Communication for Development and Gender Studies, The Angels Trust, Ymergatan 3, Box 372, SE- 101 27 Stockholm, Sweden

## Abstract

**Background:**

The Niger Delta region of Nigeria has been undergoing collective violence for over 25 years, which has constituted a major public health problem. The objectives of this study were to investigate the predictors of women's attitudes toward intimate partner violence in the Niger Delta in comparison to that of women in other parts of Nigeria.

**Methods:**

The 2003 Nigeria Demographic and Health Survey was used for this study. Respondents were selected using a stratified two-stage cluster sampling procedure through which 3725 women were selected and interviewed. These women contributed 6029 live born children born to the survey. Internal consistency of the measure of the women's attitudes towards intimate partner violence against a woman was assessed using Cronbach's alpha (α). Percentage distributions of the relevant characteristics of the respondents were carried out, and multivariable logistic regression analysis was used to measure the magnitude and direction of the relationship between the outcome and predictor variables were expressed as odds ratios (OR) and statistical significance was determined at the 95 percent confident interval level (CI).

**Results:**

Tolerance for intimate partner violence among the women in the Niger delta (47 percent) was higher than that of women from the rest of the country (42 percent). Rural residence, lower household wealth, lower status occupations, and media access (newspaper and radio) were associated with higher risk of justifying IPV among the women in the Niger Delta. In contrast full or partial autonomy in household decisions regarding food to be cooked, and access to television were associated with a lower risk of justifying violence.

**Conclusion:**

The increased justification of intimate partner violence among the women in the Niger Delta could be explained by a combination of factors, among which are cognitive dissonance theory (attitudes that do not fit with other opinions they hold as a means of coping with their situation), ecological theory (behaviour or attitudes being shaped by current factors in their neighbourhood, community or family), and gender-role attitudes. Further in-depth studies are required to fully understand women's attitudes toward violence in areas of conflict

## Background

Intimate partner violence (IPV) against women is the most common form of violence against women [1]. It is now a well known public health problem and a significant source of human rights violation worldwide [2]. Intimate partner violence against women constitutes a major risk factor to women's health, with physical and mental health consequences [3]. Though the impact of areas of conflict on women's health differs considerably between contexts and between individuals, women are generally susceptible to marginalization, poverty and suffering, with exacerbation of existing inequalities and patterns of discrimination, and violent acts [4]. Studies both in Africa and other areas of conflict indicate that domestic violence increases both during and after armed conflict [5]. This is attributed to the acceptance of violence as a means of asserting power and resolving conflicts; the changing role of women in society; lawlessness and a climate of impunity; weak or absent security provision such as effective policing, and hidden male trauma [5]. Cross-cultural studies indicate that IPV is prevalent in societies with high levels of violence and conflict and that there is an association between collective violence and interpersonal violence [6-8]. Even children raised in the midst of conflict may come to believe that violence is an appropriate way to settle interpersonal conflicts [8]. Several studies report that intimate partner violence is acceptable in some instances as justifiable punishment for a woman's transgression of her normative roles in society by significant proportions of men and women in sub-Saharan Africa [9-13], suggesting therefore that such attitudes toward violence are deeply rooted in the traditional gendered roles of women and men [1]

### Intimate partner violence in Nigeria

Intimate partner violence is pervasive in Nigeria [14-16], and its prevalence has been reported to vary between different regions and states of the country, from as low as 24% in South Western Nigeria [17], 31% in western Nigeria – Lagos [14] and Ibadan [18], 39% in Anambra State [16], to as high as 79% in Imo State [15]. The traditional Nigerian society is patriarchal; hence violence against women is frequently tolerated in communities where women are assigned an inferior role, subordinate to the male head of the family, and effectively the property of their husbands [18]. Discriminatory laws that condone certain forms of violence against women, dismissive attitudes among the Police, an inaccessible justice system, and the fact that violence against women in the home is generally regarded as belonging in the private sphere, are all contributory factors [18,19].

### Collective violence in the Niger Delta

Collective violence is defined as "the instrumental use of violence by people who identify themselves as members of a group – whether this group is transitory or has a more permanent identity – against another group or set of individuals, in order to achieve political, economic, ideological, or social objectives" [1]. Acts that constitute collective violence, such as, civil wars; terrorism; communal violence; violent political conflicts that occur within or between states; state-perpetrated violence such as genocide, repression, disappearances, torture and other human rights abuses; and organized violent crime [20], are a public health problem, and for over 25 years, unrest and violence in the Niger Delta have slowly escalated into a guerrilla-style conflict resulting in hundreds of deaths each year. Collective violence in the Niger Delta is characterized by militancy and criminality, exemplified by: oil theft (also known as "illegal bunkering"), armed violence, extortion and kidnappings by militant groups [20], extra-judicial killings by Nigeria's security forces [21,21], rampant human rights abuses by security forces intent on crushing any resistance to the regions oil production [22,23].

The conflict is mainly motivated by the ongoing struggle for the control of the crude oil and gas resources that make Nigeria the number one oil producer in Africa, the world's tenth largest crude oil producer and the fifth biggest supplier of crude imports to the United States [20]. It is also motivated by the perceived injustice of the people of the Niger Delta perpetrated against them by the Nigerian government [24]. Other triggers of the violence include: habitual lack of social development, such as, lack of roads, schools, electricity, and health services in the Niger Delta, which has resulted in violence becoming an accepted norm in that society, and educated Delta indigenes leaving the region [25]. In addition, consequences of oil spills, such as, environmental pollution, river pollution, and gas flares, severely destroy farmlands and rivers, failure of animals to thrive, and people being denied their livelihood [26], inter-ethnic violence [20,26], insecurity, lack of political freedom, anger over high levels of unemployment, and economic marginalization [27] fuel the conflict.

### The Niger Delta Context

The Niger Delta has a steadily growing population estimated in 2005 at over 30 million people, accounts for more than 23% of Nigeria's total population, and has a population density that is among the highest in the world with 265 people per square kilometer. It consists of nine States: Rivers, Bayelsa and Delta, Abia, Akwa Ibom, Cross Rivers, Edo, Imo and Ondo States [25]. Despite its natural resources accounting for over 95% of Nigeria's proven gas and oil reserves [28], the Niger Delta suffers from administrative neglect, crumbling social infrastructure and services, high unemployment, social deprivation, abject poverty, filth, squalor, and endemic conflict [29]. Studies on attitudes of women towards intimate partner violence in the Niger Delta are almost non-existent, so also are studies on attitudes toward intimate partner violence in areas of armed conflict. This study however attempts to fill that gap.

The objectives of this study were to: *i) *investigate the predictors of women's attitudes toward intimate partner violence in the Niger Delta in comparison to the attitudes of women from other parts of Nigeria. It hypothesizes that the women of the Niger Delta have a higher risk of accepting or justify violence under certain circumstances.

## Methods

### Definition

The UN Declaration on the Elimination of Violence against Women defines violence against women as ".... any act of gender-based violence that results in, or is likely to result in physical, sexual, or psychological harm or suffering to women, including threats of such acts, coercion or arbitrary deprivation of liberty, whether occurring in public or in private life" [30]. Intimate partner violence (IPV) against women is defined as "the range of sexually, psychologically, and physically coercive acts used against adult and adolescent women by current or former male partners" [31]. Intimate partner violence is often used interchangeably with violence against women and gender-based violence.

### Design and sample

Data from the 2003 Nigeria Demographic and Health Survey (DHS) was used in this study. This is a nationally representative probability sample, collected using a stratified two-stage cluster sampling procedure. Sampling of women was performed according to the list of enumeration areas developed from the 1991 Population Census sampling frame. The first sampling stage involved selecting 365 clusters (primary sampling units) with a probability proportional to the size, the size being the number of households in the cluster. The second sampling stage involved systematically selecting households from the selected clusters. This resulted in a nationally representative probability sample of 7864 households. From these households, data was collected by face-to-face interviews from 3725 women aged 15 – 49 years [32]. Of a total sample of women (*n *= 3725) in the whole survey, 771 were resident in the Niger Delta.

### Questionnaire

The comprehensive DHS questionnaire that covered issues ranging from demographic, socio-economic to health issues, as well as child health and welfare, women empowerment and social status, and husband's status was used. The current study specifically used questions in the DHS questionnaire in which respondents were asked: "sometimes a husband is annoyed or angered by things that his wife/partner does. In your opinion, is a husband justified in hitting or beating his wife in the following situations... *a) *If she goes out without telling him? *b) *If she neglects the children? *c) *If she argues with him? *d) *If she refuses to have sex with him? e) If she burns the food? and *f) *if the food is not cooked on time?"

### Specification and measurement of variables

#### Outcome variables

The outcome variable "justifies violence" was created from the five questions mentioned above. Responses to these questions were transformed into a single dichotomous "Yes" or "No" variable. Women who responded "yes" to one or several of the attitude questions formed one group of the dichotomy, were considered to be the risk group of the dichotomy, and were coded as 1. The women who responded "no" to all the attitude questions (i.e. a firm negative response) formed the other group of the dichotomy, and were coded as 0.

#### Predictor variables

The logistic regression analysis included the following predictor variables: *Demographic characteristics were *assessed using the following indicators: age (grouped as 15–18, 19–23, 24–28, 29–33, >34); marital status (grouped as "never married", "currently married", "formerly married"); ethnic affiliation assessed as a merger of Fulani/Hausa/Kanuri ethnic groups [which were categorized based on the criteria that these ethnic groups either speak a common language or dialect; share a common sense of identity, cohesion and history; have a single set of customs and behavioural rules as in marriage, clothing, diet, taboos and so on], Igbo, Yoruba, and Others (a merger of other minor ethnic groups); and religious affiliation (classified as Christian, Muslim, Traditional and others). *Socio-economic status *was assessed using the following variables: highest level of education, classified as no education, primary, secondary or higher. Occupation was assessed as: professional/technical/managerial, clerical/sales/services/skilled manual, agricultural self employed/agricultural employee/household & domestic/unskilled manual occupations; and not working. Wealth index (an indicator of the economic status of households that is consistent with expenditure and income measures) was constructed to represent the household's economic level using principal component analysis, since the DHS does not generally collect information on household income or wealth. These weighted values were then summed and rescaled to range from 0 to 1, and each household assigned to either the poorest, middle, and richest tertials.

Women's empowerment was assessed using the following indicators: (i) *autonomy in domestic decisions *was assessed by asking if the women if they had final say regarding: "large household purchases", "daily household purchases", "visits to family or friends, "own health", and "food to be cooked each day". Possible response options "respondent alone", "respondent and husband/partner", "respondent and other person in the household", formed one group of the dichotomy, while the options "husband/partner alone", and "someone else" formed the other group of the dichotomy; (ii) *access to media *was assessed using questions on frequency of listening to radio, reading newspapers/magazines, and watching television; and responses were dichotomized into "not at all" in one group or "less than once a week", "at least once a week", and "almost every day" in the other group; and (iii) *literacy level*, considered as a factor influencing access to information, was assessed as the ability to read (being "able to read whole sentences" formed one group of the dichotomy, while "able to read part of a sentence" and "unable to read" were considered as representing illiteracy, and formed the other group of the dichotomy.

### Statistical Analysis

The analyses were performed using the Statistical Package for the Social Sciences (SPSS) version 16.0 [33]. Internal consistency of the questions used to measure the women's justification of intimate partner violence against a woman was assessed using Cronbach's alpha (α). Percentage distributions of the demographic and other relevant characteristics of the respondents were carried out among the women in the Niger Delta and the rest of the women. Only the predictor variables that were statistically significant in the bivariate analyses (*p *< .05) were entered into the multivariate logistic regression models all in a single block to control for possible confounding between these variables. The magnitude and direction of the relationship between the outcome and predictor variables were expressed as odds ratios (OR) and statistical significance was determined at the 95 percent confident interval level (CI). Missing data were excluded from the analysis.

### Ethical considerations

The survey procedure and instruments for the 2003 Nigeria DHS were approved by the National Ethics Committee in the Federal Ministry of Health, Nigeria and by the Ethics Committee of the Opinion Research Corporation Macro International Incorporated (ORC Macro Inc.), Calverton, USA. Informed consent was obtained from all participants prior to participation in the survey, and collection of information was done confidentially. This study is based on analysis of secondary data with all participant identifiers removed. Ethical permission for use of the data in the present study was obtained from ORC Macro Inc.

## Results

### Justifying wife beating

**Figure 1:**
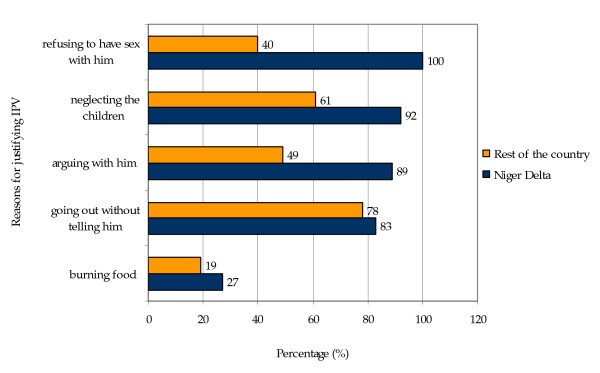
**Percentage distribution of women's reasons for justifying intimate partner violence in the Niger delta**.

Wife beating was widely accepted under several circumstances by the women in the sample. 47 percent of women in the Niger Delta would justify IPV for at least one of the given reasons, while 42 percent of the women in the rest of country would justify IPV for at least one of the reasons. Women in the Niger Delta would justify IPV after: going out without telling him (83%), neglecting the children (92%), arguing with him (89%), refusing to have sex with him (1000%), and burning food (27%). The women in the rest of the country would justify IPV for reasons such as, going out without telling him (78%), neglecting the children (61%), arguing with him (49%), refusing to have sex with him (40%), and burning food (19%). The percentage distribution of women who justify wife beating is justified is presented in Figure 1.

The reliability coefficients of the variables measuring acceptance of intimate partner violence using Cronbach's alpha was 0.88.

#### Proportion of the sample of women with tolerant attitudes toward wife beating by predictor variables

Of the women in the sample, the majority of the women from the Niger Delta that justified IPV were below 33 years of age (77 percent), currently married (95 percent), rural residents (77 percent), Muslims (63 percent), uneducated (59 percent), and poor (51 percent) (Table 1). Most (49 percent) of the women that justified IPV were of the Hausa/Fulani/Kanuri ethnic groups, and clerical, sales, services, skilled manual employees (41 percent). The majority of the women that justified IPV did not participate in decision-making in the household regarding: own health (81 percent), large household purchases (83 percent), purchases of daily needs (71 percent), visits to family or relatives (64 percent), and food to be cooked (60 percent). Finally, a large majority of the women in the Niger Delta were illiterate (80 percent), did not have access to newspapers or magazines (93 percent), and television (70 percent). However, majority (67 percent) of the women that justified IPV listened to the radio.

**Table 1:** Proportion of women in the Niger Delta with tolerant attitudes toward violence by predictor indicators

Variables	Niger Delta		Other women	
	
	*n*	(%)	*n*	(%)
***Demographic characteristics***				
*Age*				
15 – 18	23	5	102	4
19–23	97	21	449	20
24–28	143	31	687	31
29–33	92	20	450	20
34+	105	23	59	25
*Marital status*				
Never married	5	1	24	1
Currently married	436	95	2158	96
Formerly married	19	4	65	3
*Place of residence*				
Urban	107	23	789	35
Rural	353	77	1458	65
*Ethnic affiliation*				
Hausa/Fulani/Kanuri	225	49	1169	52
Igbo	18	4	154	7
Yoruba	26	6	250	11
Others	191	41	674	30
*Religious affiliation*				
Christian	160	35	704	31
Islam	292	63	1419	63
Traditional/other	8	2	51	2
***Socio-economic characteristics***				
*Highest level of education*				
No education	272	59	1265	56
Primary	115	25	519	23
Secondary or higher	73	16	463	21
***Occupation***				
Professional/Technical/Management	9	2	70	3
Clerical, sales, services, skilled manual	189	41	1026	46
Agric. self., Agric. employee, household & domestic, unskilled manual	96	21	382	17
Not working	166	36	769	34
*Wealth index*				
Poor	234	51	737	33
Middle	182	40	746	33
Rich	44	9	764	33
***Decision-making autonomy***				
*Final say on own health*				
Not at all	373	81	1864	83
Full or partial	87	19	371	17
*Final say on large household purchases*				
Not at all	381	83	1876	84
Full or partial	79	17	351	16
*Final say on household purchases for daily needs*				
Not at all	327	71	1602	72
Full or partial	133	29	630	28
*Final say on visits to family or relatives*				
Not at all	294	64	1485	67
Full or partial	166	36	741	33
*Final say on food to be cooked*				
Not at all	275	60	1240	56
Full or partial	185	40	990	44
***Access to media***				
*Reads newspaper or magazine*				
Not at all	427	93	1959	88
Yes	27	7	276	12
*Listens to radio*				
Not at all	154	33	544	24
Yes	306	67	1701	76
*Watches television*				
Not at all	324	70	1475	66
Yes	136	30	772	34
***Literacy level***				
*Literacy*				
Cannot read/cannot read fully	364	80	1617	73
Can read fully	93	20	602	27

Of the women in the sample from the rest of the country that justified IPV, majority (75 percent) were below 33 years of age, currently married (96 percent), rural resident (65 percent), Hausa/Fulani/Kanuri (52 percent), Muslims (63 percent), and illiterate (56 percent). In addition, most of the women from the rest of the country that justified IPV were without autonomy in household decisions regarding own health (83 percent), large household purchases (84 percent), purchases for daily needs (72 percent), visits to family or relatives (67 percent), and food to be cooked (56 percent). Finally, majority of the women from the rest of the country that justified IPV were illiterate (73 percent), did not have access to newspaper (88 percent), and television (66 percent). However, a higher proportion of the women who justified IPV had access to radio (76 percent).

#### Associations between predictor variables and acceptance of IPV among the sample of women in the Niger Delta and the rest of the country toward IPV

Among the women in the Niger Delta, rural residents were at higher risk of justifying IPV compared to urban residents (OR = 1.37, 95% CI = 1.05 – 1.78) (Table 2). Women from poor (OR = 3.25, 95% CI = 2.06 – 5.13), and middle (OR = 2.19, 95% CI = 1.47 – 3.27) wealth tertials were at three times and twice higher risk of justifying IPV compared to women in the rich wealth tertial respectively. In addition, women who had full or partial autonomy in household decisions regarding food to be cooked (OR = 0.76, 95% CI = 0.60 – 0.97) had a lower risk of justifying IPV compared to women who did not have autonomy. Women who did not have access to newspapers or magazines had twice as high risk of justifying IPV as women who had access (OR = 2.19, 95% CI = 1.39 – 3.51), and women who had access to radio had a higher risk of justifying violence compared to women who had no access to radio (OR = 1.32, 95% CI = 1.05 – 1.66). Access to television resulted in the women having a lower risk of justifying IPV (OR = 0.63, 95% CI = 0.48 – 0.83) compared to women who had no access to television.

**Table 2:** Logistic regression analysis of the attitudes toward IPV by predictor variables, among the women in the sample, with Odds ratios (OR) with confidence interval (CI)

	Niger Delta	Rest of the women
**Variables**	**OR(95% CI)**	**OR(95% CI)**

*Place of residence*		
Urban	1.00	ns
Rural	1.37 (1.05 – 1.78)	
*Religious affiliation*		
Christian	1.00	ns
Islam	1.33 (1.00 – 1.77)	
Traditional/other	0.63 (0.29 – 1.35)	
*Ethnic affiliation*		
Hausa/Fulani/Kanuri	ns	1.00
Igbo		0.53 (0.42 – 0.67)
Yoruba		0.95 (0.76 – 1.18)
Others		0.76 (0.67 – 0.88)
*Highest level of education*		
No education	0.939 (0.51 – 1.74)	ns
Primary	0.958 (0.56 – 1.64)	
Secondary or higher	1.00	
***Occupation***		
Professional/Technician/Management	1.00	1.00
Clerical, sales, services, skilled manual	0.94 (0.46 – 1.95)	1.28 (0.92 – 1.77)
Agric. self., Agric. employee, household & domestic, unskilled manual	1.07 (0.50 – 2.30)	1.95 (1.37 – 2.79)
Not working	0.99 (0.47 – 2.07)	1.20 (0.86 – 1.67)
*Wealth index*		
Poor	3.25 (2.06 – 5.13)	0.84 (0.69 – 1.02)
Middle	2.19 (1.47 – 3.27)	0.90 (0.75 – 1.08)
Rich	1.00	1.00
*Final say on own health*		
Not at all	1.00	1.00
Full or partial	0.82 (0.61 – 1.10)	0.67 (0.55 – 0.81)
*Final say on making large household purchases*		
Not at all	ns	1.00
Full or partial		1.25 (1.03 – 1. 53)
*Final say on household purchases for daily needs*		
Not at all	1.00	ns
Full or partial	1.06 (0.80 – 1.40)	
*Final say on food to be cooked*		
Not at all	1.00	ns
Full or partial	0.76 (0.60 – 0.97)	
*Final say on visits to family or friends*		
Not at all	ns	1.00
Full or partial		0.80 (0.70 – 0.92)
*Reads newspaper or magazine*		
Not at all	1.00	ns
Yes	2.19 (1.37 – 3.51)	
*Listens to radio*		
Not at all	1.00	1.00
Yes	1.32 (1.05 – 1.66)	1.60 (1.40 – 1.83)
*Watches television*		
Not at all	1.00	1.00
Yes	0.63 (0.48 – 0.83)	1.10 (0.93 – 1.31)
*Literacy level*		
Cannot read/cannot read fully	1.00	ns
Can read fully	1.17 (0.70 – 1.95)	

Abbreviations: OR = Odds ratio; CI = Confidence interval; ns = not significant in bivariate analysis

Data source: 2003 Nigeria Demographic and Health Survey

Only variables that were significantly associated with men's attitudes towards IPV in the bivariate analyses were inputted into the multivariate regression model.

Among the women from the rest of the country, Igbo women (OR = 0.53, 95% CI = 0.42 – 0.67) and women from the Other ethnic groups (OR = 0.76, 95% CI = 0.67 – 0.88) were at lower risk of justifying IPV than Hausa/Fulani/Kanuri women respectively. Working as Agric. self-employed, Agric. employee, household & domestic, unskilled manual workers (OR = 1.95, 95% CI 1.37 – 2.79) were statistically significant risk factors for justifying IPV compared to working as Professional/Technician/Management workers. In addition, women in the poor (OR = 0.84, 95% CI = (0.69 – 1.02) and middle (OR = 0.90, 95% CI = 0.75 – 1.08) wealth tertials had lower risks of justifying IPV compared to women in the rich tertial. Women who had full of partial autonomy in household decisions regarding decisions about own health (OR = 0.67, 95% CI = 0.55 – 0.81) and visits to family or friends (OR = 0.80, 95% CI = 0.70 – 0.92) had a lower risk of justifying IPV compared to women who lacked autonomy. In contrast, women who had full autonomy regarding decisions about making large household purchases (OR = 1.25, 95% CI = 1.03 – 1.53) had a higher risk of justifying IPV than women who lacked autonomy. Finally, listening to radio signified a higher risk of justifying IPV (OR = 1.60, 95% CI = 1.40 – 1.83) compared to not listening at al among the rest of the women. The reliability coefficients of the variables measuring decision-making autonomy using Cronbach's alpha was 0.83.

## Discussion

Intimate partner violence is generally tolerated under several circumstances among women in the Niger Delta and among women in the rest of country. Of the women in the Niger Delta, 47 percent would justify IPV in at least one of the examined reasons, and among the women in the rest of the country, 42 percent would justify IPV for at least one of the above-mentioned reasons. More women in the Niger Delta than in the rest of the country would justify IPV for all the following reasons: going out without telling him, neglecting the children, arguing with him, refusing to have sex with him, and burning food (Figure 1).

Though comparable studies are lacking in other regions of conflict in Africa, the results of this study suggest that rural residence, lower household wealth, and lower status occupations (Agriculture self-employed., Agriculture employee, household & domestic, unskilled manual) of the women in the Niger Delta was associated with a higher risk of justifying IPV. Similar findings of tolerant attitudes of women with lower household wealth towards violence in non-conflict areas have also been reported in other studies [34,35]. This is as expected since women with low socio-economic status are likely to experience violence due to their limited resources [35]. In the Niger Delta, the origins of collective violence is rooted in a combination of factors like unemployment, poverty, deprived livelihood, absent social amenities, and dissatisfaction with the central government. These factors increase both the women's risk of being victimized, and their risk of justifying violence against them – point corroborated by Heise, 1998 [36]. Possible theoretical explanations for these acceptance attitudes could include the cognitive dissonance theory, which refers to the distressing mental state in which individuals feel "they find themselves doing things that don't fit with what they know, or having opinions that do not fit with other opinions they hold [37]. These women find themselves trapped in an environment of conflict, and as such, tend to cope with, and make sense of their experiences by accepting violence toward them in certain circumstances. This theory could also explain why some women accept or justify intimate partner violence against them. Another plausible explanation for the women's attitude of justifying IPV could be the ecological theory, which is based on multiple, interconnected elements of individuals, communities, institutions, and cultures, and suggests that an individual's behaviour is shaped not only by his/her upbringing, but by current contextual factors such as the batterer, reactions he/she receives from those around him/her, and the resources available to him/her [37,38]. This two-way interplay includes the family, the neighbourhood or workplace, the broader social influence of the media, and ideologies and/or law [39]. The attitude of the women may have therefore been shaped by current contextual factors (armed conflict in the region, low socio-economic status) around them and the resources available to them.

The association between having full or partial autonomy in household decisions regarding food to be cooked and a lower risk of justifying violence is in agreement with findings from other studies [10,40]. It underscores the importance of women's empowerment through decision-making, as interventions that promote joint decision-making might influence men and women's views towards equality in marriage and encourage men to settle household disputes with negotiation, rather than violence. Deeply entrenched social beliefs regarding the subordinate roles of women constitute a strong barrier to preventing acceptance of violence against women. This warrants the implementation of positive action to change these traditional attitudes through systematic and comprehensive education and awareness programmes, including educating women and girls about their right to live free of violence. Furthermore, the causes of the conflict in the region need to be appropriately addressed. Since the militant groups in the delta are connected to the communities, unless both state and federal governments seriously address grievances of the people of the Niger delta such as, environmental degradation, wide-spread poverty, under-development, and corruption, conflict in the delta will continue, with women and children bearing the brunt.

The association between access to media and the risk of acceptance to violence is conflicting. The increased risk of acceptance of violence amongst women with access to newspaper and radio is worthy of note, and is in contrast to findings from other studies [13,34]. This may be linked with the coverage of the violence itself in the more "independent" newspapers and radio stations (in contrast to the more "regulated" coverage by television stations) since media coverage of conflicts plays a large role in sensationalizing people, and thus exacerbating conflicts [40].

Subsequent efforts towards peace in areas of collective violence should include working with the media on their reporting of conflicts. Access to television was associated with a lower risk of acceptance of violence. The explanation for this finding is unclear, and needs to be researched further; however, it may be linked with the more controlled coverage of the conflict in the Niger Delta by the television stations, which has been described as episodic or sporadic reporting about the conflict only when the crises are exacerbated [41].

Among the women from the rest of the country, women from the Igbo and Other ethnic groups had a lower risk of justifying IPV. This may be associated with the gender-restrictiveness of the Hausa/Fulani/Kanuri ethnic groups that predisposes these women to IPV [13,42,43]. Women in lower status occupations (Agriculture self-employed, Agriculture employee, household & domestic, unskilled manual) from the rest of the country had a 95 percent higher risk of justifying IPV compared to women with higher status occupations (Professional/Technician/Management). This is a common finding, given that woman with low socio-economic status are predisposed to experiencing violence due to their limited resources [34,35]. Lower risk of justifying IPV among women with full or partial autonomy in decisions regarding own health, and visits to family or friends is generally associated with women's increased empowerment [17,41]. However, higher risk of justifying IPV among women with full or partial autonomy in decisions regarding making large household purchases is conflicting, and needs to be researched further [41]. The situation in the Niger Delta is complex, and is worsened in part by the traditional/cultural gender ideologies in Nigeria, which tend to have a strong hold on the women. Changing attitudes toward violence in the Niger Delta requires long-term commitment and strategies involving all parts of society. This would initially require the resolution of the conflict, and subsequently, stronger commitments by governments in passing and enforcing laws that ensure women's legal rights and punish abusers. Furthermore, community-based strategies can focus on empowering women, reaching out to men, and changing the beliefs and attitudes that permit abusive behaviour. Only when women are treated as equal members of society will attitudes toward violence, and violent acts against women change from being an invisible norm into a shocking aberration.

## Conclusion

This study set out to investigate the predictors of women's attitudes toward intimate partner violence in the Niger Delta (a region undergoing escalating collective violence) in comparison with that of women from the rest of the country. Residence in rural areas, lower household wealth and occupational status, access to newspaper and radio, were associated with a higher risk of justifying violence among the women. The women's increased risk of justifying violence could be generally explained by a combination of theories, such as the cognitive dissonance theory (women having opinions or attitudes that do not fit with other opinions they hold as a means of coping with their situation), and ecological theory (women's behaviour or attitudes being shaped by current factors in their neighbourhood, community or family.

## Competing interests

The authors declare that they have no competing interests.

## Authors' contributions

Both authors have read and approved the final manuscript. DA: Major role in study conception, data extraction, analyses, and writing of the manuscript. JA: Study conception, discussion, and manuscript writing

## Pre-publication history

The pre-publication history for this paper can be accessed here:

http://www.biomedcentral.com/1472-698X/9/12/prepub
